# *Daphne jejudoensis* Attenuates LPS-Induced Inflammation by Inhibiting TNF-α, IL-1β, IL-6, iNOS, and COX-2 Expression in Periodontal Ligament Cells

**DOI:** 10.3390/ph15040387

**Published:** 2022-03-22

**Authors:** Ji-Yeong Bae, Dong-Seol Lee, You Kyoung Cho, Ji-Yeon Lee, Joo-Hwang Park, Sang Ho Lee

**Affiliations:** 1 College of Pharmacy and Jeju Research Institute of Pharmaceutical Sciences, Jeju National University, Jeju 38655, Korea; jybae@jejunu.ac.kr; 2 Interdisciplinary Graduate Program in Advanced Convergence Technology & Science, Jeju National University, Jeju 63243, Korea; alwaysnewly@gmail.com; 3 R&D Center, Regenerative Dental Medicine Institute, HysensBio Co., Ltd., Gwacheon 13814, Korea; dslee@hysensbio.com (D.-S.L.); jyk4335@hysensbio.com (Y.K.C.); orange@hysensbio.com (J.-H.P.)

**Keywords:** *Daphne jejudoensis*, anti-inflammatory activity, Thymelaeaceae, periodontitis, lipopolysaccharide

## Abstract

Periodontitis is a common disease involving inflammation and tissue destruction in the periodontal region. Although uncontrolled long-term inflammation in the gingiva may lead to loss of the periodontal ligament, treatments or preventive solutions for periodontitis are scarce. The aim of this study is to find anti-inflammatory material from a natural source that can be used to treat or protect against periodontitis. *Daphne* species (Thymelaeaceae) are important and popular components of traditional Chinese medicine and are used as anti-inflammatory agents. *Daphne jejudoensis* is an endemic plant that grows on Jeju Island and was identified as a new species in 2013. In this study, for the first time, we investigated the anti-inflammatory effect of *D. jejudoensis* leaf extract (DJLE) on human periodontal ligament cells. The gene expression levels of pro-inflammatory cytokines (interleukin-1β and 6 and tumor necrosis factor-α) and inflammation-inducible enzymes (inducible nitric oxide synthase and cyclooxygenase-2) were reduced after DJLE treatment with/without lipopolysaccharide stimulation. The findings of this study indicate that *D. jejudoensis* possesses anti-inflammatory activities, suggesting that DJLE may be a potential preventive and therapeutic agent for periodontitis.

## 1. Introduction

Periodontal disease, gingivitis, and periodontitis are chronic inflammatory diseases caused by periodontal pathogenic bacteria that compromise the integrity of tooth-supporting tissues [1]. Gingivitis, a mild form of periodontal disease, is a reversible condition in which inflammatory cell infiltrates are restricted within the gingival epithelium and connective tissue. In contrast to gingivitis, periodontitis is irreversible and represents an extensive inflammatory attack that causes progressive degradation of the cementum, periodontal ligament, and alveolar bone, resulting in tooth loss [2]. The continuous loss of teeth owing to periodontitis seriously affects quality of life and causes social and economic burdens [3]. According to the results of the Global Burden of Disease study, severe periodontitis is one of the most prevalent chronic inflammatory diseases worldwide [4], affecting approximately 10% of the global adult population [5,6]. Moreover, clinical and experimental evidence has revealed that periodontitis increases the risk of other disorders, such as diabetes, rheumatoid arthritis, atherosclerosis, and Alzheimer’s disease [7,8,9]. The local microbiota and host immune response are related to the initiation and progression of periodontitis, and signaling molecules and cytokines are important modulators of both homeostasis and inflammatory processes [10,11]. Many studies have shown that disordered regulation of cytokines initiates periodontitis [12,13,14,15] and that manipulating cytokine expression reduces alveolar bone loss [16,17]. Patients with an ulcerated gingival epithelium and severe periodontitis show elevated cytokine levels. Periodontal pathogens, such as *Porphyromonas gingivalis*, *Treponema denticola*, *Prevotella intermedia*, and *Aggregatibacter actinomycetemcomitans*, produce a bacterial membrane protein called lipopolysaccharide (LPS) [18,19]. LPS stimulates inflammatory cells to induce high levels of proinflammatory cytokines, such as interleukin (IL)-1β, IL-6, and tumor necrosis factor-alpha (TNF-α), which causes periodontal tissue destruction [20]. In addition, pro-inflammatory cytokines produced in human periodontal ligament cells (hPDLCs) may play an important role in periodontal disease [20,21]. Nonsteroidal anti-inflammatory drugs (NSAIDs) are widely used in the treatment of periodontal disease [22,23,24]. However, owing to the side effects of NSAIDs, it is important to discover alternative medicines with fewer side effects, such as plant-derived anti-inflammatory agents.

As part of our ongoing research project on discovering anti-inflammatory materials against periodontitis from endemic natural resources, we came to find the anti-inflammatory effect of *Daphne jejudoensis* leaf extract (DJLE) on hPDLCs while screening TNF-α reduction activity using several plant extracts. *Daphne jejudoensis* (Thymelaeaceae) is native to Jeju Island in South Korea, especially in the Gotjawal forest areas, such as Seonheul, Andeog, and Mureung. Despite its similarity to *D. kiusuana*, it was reported as a new species in 2013 owing to a number of morphological differences, such as glabrous calyx surface, long calyx tube and lobes, elliptic leaves with acuminate apex, and unique distribution area in the forest region on Jeju Island [25,26]. Thus, it can be distinguished from *D. kiusiana,* which has hairy calyx tube and lobes, short calyx lobe, and oblanceolate leaves with acute apex [26].

*Daphne* comprises over 70 species that are widely distributed in temperate climate zones, including Europe, Asia, and North America. Owing to their pleasant, fragrant flowers, the major usage of these genera is as garden trees [27]. *Daphne* contains various classes of phytochemicals, including coumarins, flavonoids, terpenes, steroids, and lignans [2]. Several species of *Daphne* have been used in traditional medicine, especially in Asia, owing to their anti-inflammatory, diuretic, anticancer, analgesic, antioxidant, antitussive, and immunomodulatory activities [28,29,30]. Genkwadaphnin, 1,2-dehydrodaphnetoxin, and daphnetin from *D. oleoides* ssp. *oleoides* showed inhibitory effects on the synthesis of IL-1α, IL-1β, and TNF-α, supporting the evidence for folkloric use in rheumatoid arthritis [31]. Daphnoretin, isolated from the flower buds of *D. genkwa* (Genkwa Flos) and traditionally used for asthma and edema and as a diuretic [32], exhibits significant complementary modulation ability to target inflammatory disorders [30].

Although *Daphne* species have been widely used in traditional medicine, their anti-inflammatory responses against periodontal diseases have not been reported. In this study, for the first time, we investigated the anti-inflammatory effects of DJLE on LPS-induced hPDLCs by evaluating the inhibition of IL-1β, IL-6, TNF-α, inducible nitric oxide synthase (iNOS), and cyclooxygenase (COX)-2 expression.

## 2. Results

### 2.1. Cytotoxicity of DJLE on hPDLCs

**Figure 1:**
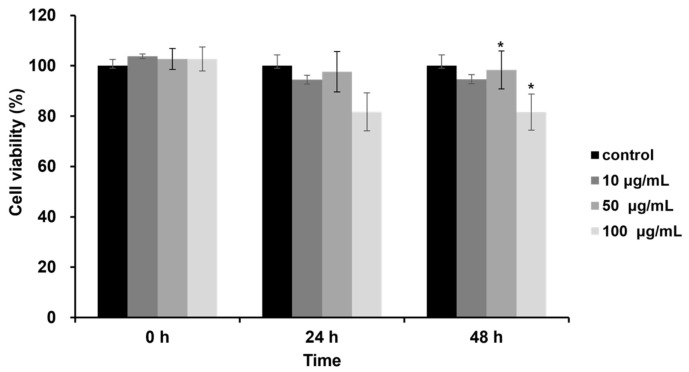
Effects of *Daphne jejudoensis* leaf extract (DJLE) on the viability of human periodontal ligament cells (hPDLCs). The cells grown in serum-free medium were treated with different concentrations (10, 50, and 100 μg/mL) of DJLE for 24 and 48 h using the MTT assay. Cell viability was expressed as the average of experiments relative to the untreated control (100%). * *p* < 0.05 vs. control.

The cytotoxicity of DJLE on hPDLCs was evaluated using the methyl thiazol-2-yl-2, 5-diphenyl tetrazolium bromide (MTT) assay. hPDLCs were treated with DJLE at concentrations of 10, 50, and 100 μg/mL (Figure 1). Cell viability was examined after 24 and 48 h of incubation and compared with that of the control group. The results showed that DJLE was not cytotoxic to treated cells (Figure 1). At high concentrations, cell viability remained above 80% in hPDLCs after 48 h of incubation.

### 2.2. Development of an In Vitro Inflammatory Cell Model Using LPS on hPDLCs

**Figure 2:**
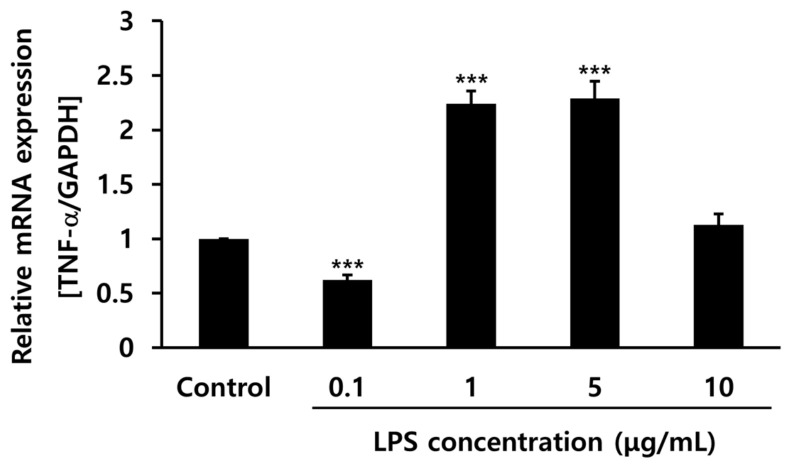
Tumor necrosis factor (TNF)-α gene expression levels with different concentrations of lipopolysaccharide (LPS) (0.1, 1, 5, and 10 μg/mL) in human periodontal ligament cells. *** *p* < 0.001 vs. control.

The optimal concentration of LPS was determined by subjecting cells to several levels of LPS treatment to develop an in vitro inflammatory cell model. A main component of the Gram-negative bacterial cell wall, LPS induces inflammation and immune responses in mammals [33]. The recognition of LPS by the innate immune system via toll-like receptor-4 leads to secretion of the pro-inflammatory cytokine TNF-α [34]. The gene expression levels of TNF-α were compared using reverse transcription–polymerase chain reaction (RT-PCR) in hPDLCs after treatment with several concentrations of LPS. At concentrations of 1 and 5 μg/mL, TNF-α gene expression increased twofold compared to that in the control. LPS (1 μg/mL) was chosen in the following experiment for the evaluation of anti-inflammatory effects (Figure 2).

### 2.3. Determination of the Optimal DJLE Concentration for the Experiment on hPDLCs

**Figure 3:**
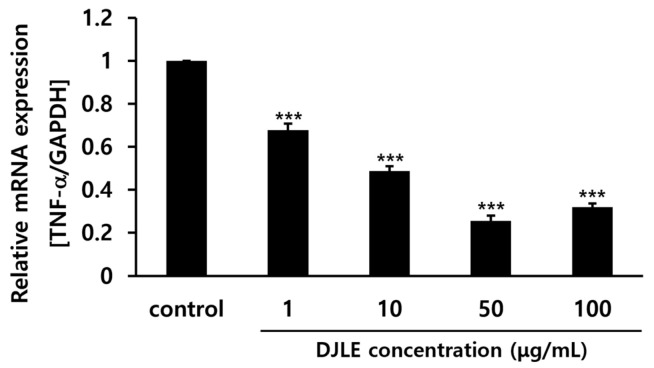
Tumor necrosis factor (TNF)-α gene expression levels in human periodontal ligament cells with different concentrations of *Daphne jejudoensis* leaf extract (DJLE) (1, 10, 50, and 100 μg/mL). *** *p* < 0.001 vs. control.

The DJLE concentration in this experiment was determined by comparing TNF-α gene expression levels in hPDLCs after treatment with different levels of DJLE. DJLE inhibited TNF-α gene expression in hPDLCs (Figure 3) in a dose-dependent manner. At a concentration of 50 μg/mL DJLE, TNF-α gene expression levels decreased by over fivefold compared to that in the control. Therefore, the optimal concentration for the DJLE anti-inflammatory experiment was set at 50 μg/mL.

### 2.4. Effect of DJLE on the Expression of TNF-α in LPS-Induced hPDLCs

**Figure 4:**
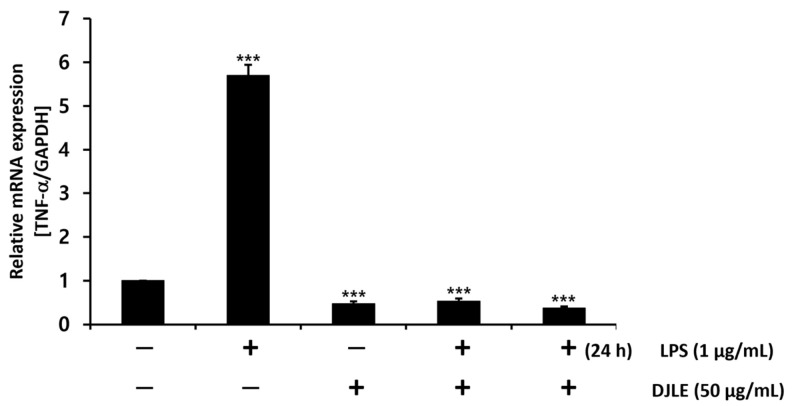
Effect of *Daphne jejudoensis* leaf extract (DJLE) on tumor necrosis factor (TNF)-α gene expression under LPS-induced inflammation in human periodontal ligament cells. *** *p* < 0.001 vs. control.

With the optimal concentration of DJLE for the evaluation of anti-inflammatory effects set at 50 μg/mL, DJLE was applied to LPS-induced inflammatory cell models. TNF-α gene expression in hPDLCs (Figure 4) was measured under different conditions as follows: (1) sham control, (2) LPS-treated group (inflammation-induced negative control), (3) DJLE single treatment (50 μg/mL), (4) LPS (1 μg/mL) + DJLE (50 μg/mL) (preventive effect), (5) LPS (1 μg/mL, 24 h pretreatment) + DJLE (50 μg/mL) (treatment effect). The negative control, i.e., the LPS-treated group, showed fivefold higher TNF-α expression than the sham control, and the DJLE single-treated group exhibited fivefold lower TNF-α gene expression than the LPS-treated group. DJLE was administered to LPS-induced cells simultaneously and 24 h after LPS injection to evaluate the preventive and therapeutic effects of DJLE, respectively. TNF-α gene expression was significantly suppressed in hPDLCs (Figure 4). Therefore, DJLE is considered to have therapeutic and preventive effects on periodontitis in vitro.

### 2.5. Effect of DJLE on the Expression of IL-1β and IL-6 in LPS-Induced hPDLCs

**Figure 5:**
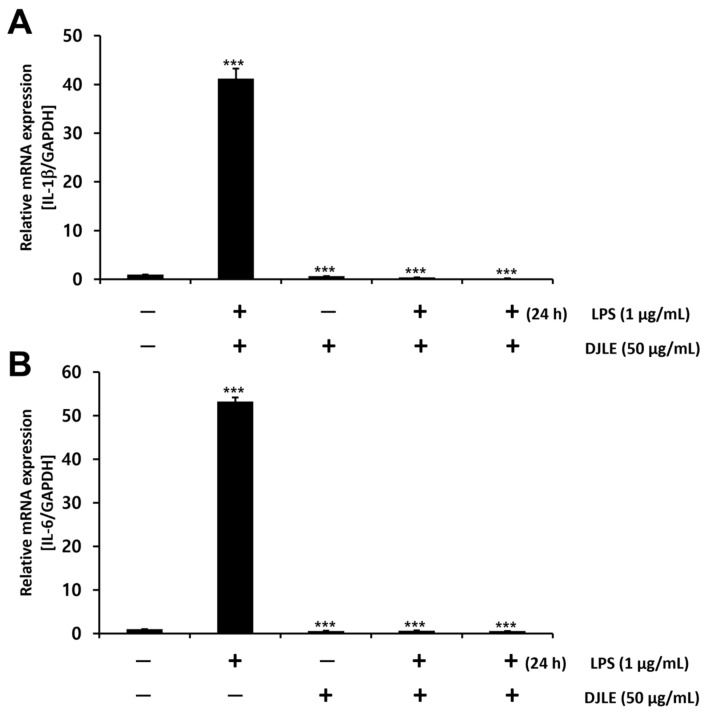
Effect of *Daphne jejudoensis* leaf extract (DJLE) on the gene expression of the proinflammatory cytokines interleukin-1β (**A**) and interleukin-6 (**B**) in lipopolysaccharide (LPS)-stimulated human periodontal ligament cells. *** *p* < 0.001 vs. control.

LPS-induced hPDLCs were treated with DJLE to evaluate its anti-inflammatory properties in terms of IL-1β and IL-6 expression levels. IL-1β and IL-6 expression levels followed a trend similar to the decreasing pattern of TNF-α gene expression after DJLE treatment. The gene expression levels of IL-1β (Figure 5A) and IL-6 (Figure 5B) in the DJLE single-treated group, LPS + DJLE simultaneously treated group (preventive effect), and LPS 24 h pretreatment + DJLE treated group (treatment effect) significantly decreased compared to the sham control and LPS-treated groups.

### 2.6. Effect of DJLE on the Expression of iNOS and COX-2 in LPS-Induced hPDLCs

**Figure 6:**
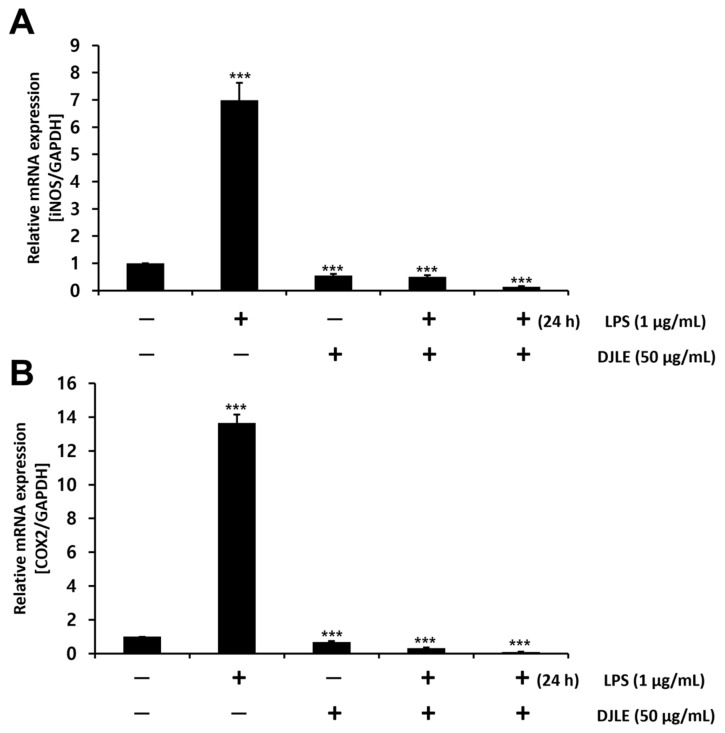
Effect of *Daphne jejudoensis* leaf extract (DJLE) on the gene expression of inducible nitric oxide synthase (iNOS) (**A**) and cyclooxygenase (COX)-2 (**B**) in lipopolysaccharide (LPS)-stimulated human periodontal ligament cells. *** *p* < 0.001 vs. control.

In macrophages, iNOS is induced by pro-inflammatory cytokine response and bacterial LPS [35]. COX transfers arachidonic acid to prostaglandins and plays an important role as a facilitator of inflammatory reactions [36]. To investigate the anti-inflammatory effects of DJLE, the gene expression levels of iNOS and COX-2 were examined and compared with those of the control in LPS-induced hPDLCs. Both iNOS (Figure 6A) and COX-2 (Figure 6B) expression levels were remarkably reduced in the DJLE alone, LPS + DJLE, and LPS 24 h pretreatment + DJLE groups.

### 2.7. Antimicrobacterial Effect of DJLE on Porphyromonas gingivalis

**Figure 7:**
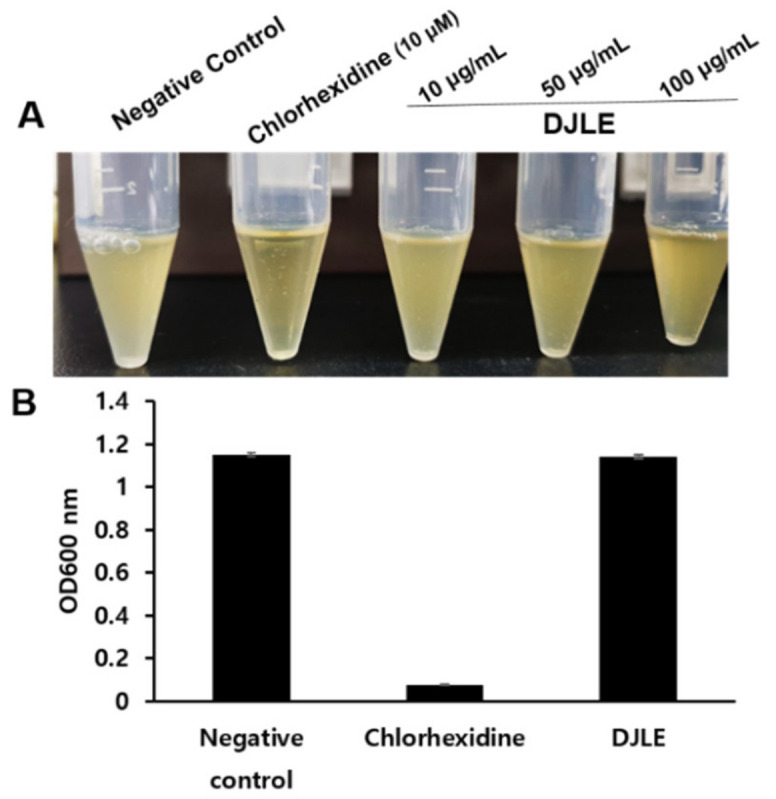
Growth inhibition effect of *Daphne jejudoensis* leaf extract (DJLE) against *Porphyromonas gingivalis*: the broth turbidity comparison with positive control, chlorhexidine (**A**), and the optical density at 600 nm (**B**).

To investigate the potential use of DJLE as an antimicrobial agent, the antimicrobial activity of DJLE against *P. gingivalis* was evaluated. DJLE was tested for its ability to inhibit *P. gingivalis* growth. DJLE was administered at different concentrations (10, 50, and 100 μg/mL) to *P. gingivalis* cultures (Figure 7A). Chlorhexidine (positive control) showed antimicrobial activity at a concentration of 10 μM (Figure 7A,B). However, DJLE (100 μg/mL) did not inhibit growth (Figure 7B).

### 2.8. Identification of Major Compounds in DJLE

**Figure 8:**
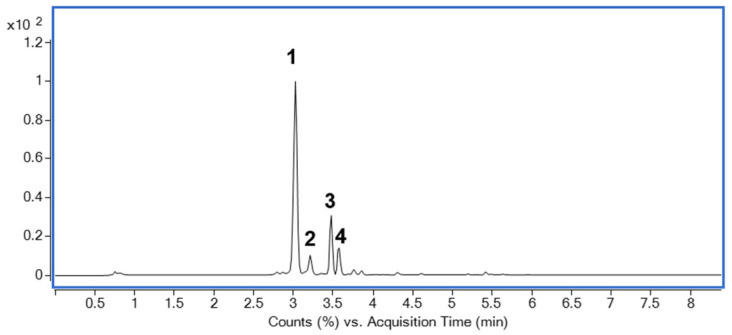
Base peak chromatogram of *Daphne jejudoensis* leaf extract (DJLE) using LC-QToF in negative ESI mode: daphnin (**1**), rutin (**2**), daphnetin (**3**), and cynaroside (**4**).

**Figure 9:**
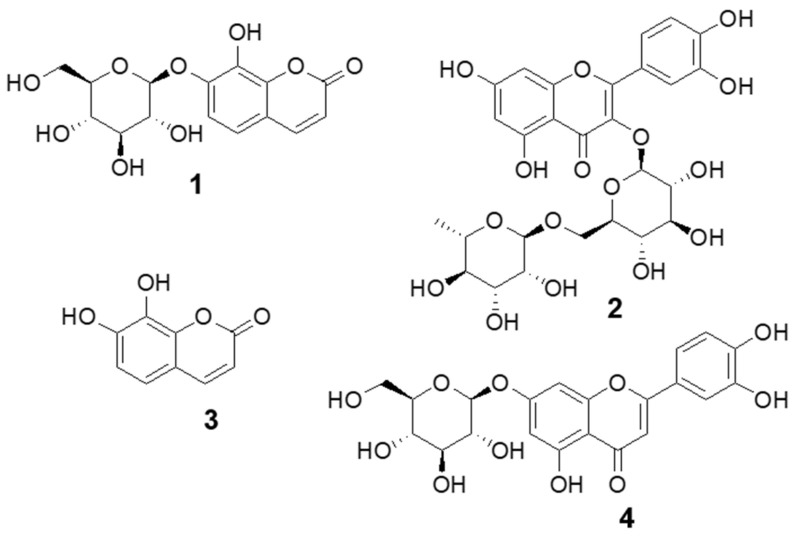
Structure of compounds identified from *Daphne jejudoensis* leaf extract (DJLE): daphnin (**1**), rutin (**2**), daphnetin (**3**), and cynaroside (**4**).

The base peak chromatogram of DJLE was obtained using an ultra-high-performance liquid chromatography system coupled with quadrupole time-of-flight (LC-QToF) mass spectrometry (Figure 8). A major component found at a retention time of 3 min was isolated using medium-pressure liquid chromatography with a mobile phase comprising a mixture of methylene chloride and methanol. The molecular formula of compound **1** was determined to be C_15_H_16_O_9_. The chemical structure was determined to be that of daphnin through its negative mode *m*/*z* 339.07162 [M-H]^−^ and mass fragment pattern of sugar loss (*m*/*z* 177.01857 [M-H-Glc]^−^) [37,38,39]. Compound **1** was confirmed to be daphnin by comparison with the retention time of the reference compound. The content of the major compound, daphnin, was calculated as 7.4 mg/g DW using the external standard method. Compound **2** had *m*/*z* 609.1456 [M-H]^−^ as a molecular ion peak, suggesting a molecular formula of C_27_H_30_O_16_, and was estimated to be rutin [40]. Compounds **3** and **4**, which showed [M-H]^−^ ions at *m*/*z* 177.0195 and *m*/*z* 447.0931, were identified as daphnetin [41] and cynaroside [42], respectively. The structures of the identified compounds are shown in Figure 9. The measured mass error was <2 ppm (mDa).

## 3. Discussion

Periodontitis is a destructive inflammatory condition caused by various periodontal bacteria that activate pro-inflammatory signaling mediators [43]. Although various medical treatments, including NSAIDs, are available, the development of anti-inflammatory products from natural resources has been of constant interest in research focused on preventing the adverse effects of NSAIDs. Plant materials previously reported for the prevention and treatment of periodontitis include sage leaves, oak bark, peppermint leaves, calamus rhizome, Baikal skullcap root, pomegranate, tea leaves, aloe vera gel, chamomile flowers, magnolia bark, blackberry leaves and fruits, cranberry fruit, and *Lippia sidoides* [2]. The aim of this study was to investigate the anti-inflammatory effects of DJLE by evaluating the inhibition of IL-1β, IL-6, TNF-α, iNOS, and COX-2 expression in hPDLCs to examine the potential to develop a treatment agent for periodontitis.

Recently, several phytochemicals isolated from *Daphne* species have been reported to exert anti-inflammatory effects against chronic obstructive pulmonary disease [34], neuroinflammation [44], and rheumatoid arthritis [2]. Genkwanin from *D. genkwa* flowers is the most active anti-rheumatoid-arthritis flavonoid. Daphnodorin C from *D. kiusiana* reduced lung inflammation via NF-κB, CREB, JNK, and p38 signaling inhibition and has been suggested as a promising treatment agent for chronic obstructive pulmonary disease [34]. (−)-Aptosimon, a lignan from the flower buds of *D. genkwa*, reduced TNF-α production and expression of COX-2 and iNOS in LPS-stimulated RAW 264.7 macrophages [35].

Coumarins isolated from stem bark of *D. feddei*, 7-hydroxycoumarin and daphnetin, showed potent inhibitory effects against nitric oxide production in LPS-induced RAW 264.7 cells [45]. The anti-inflammatory effect of DJLE may be attributed to daphnetin (from daphnin with sugar loss), which is known to be an active compound responsible for the analgesic and anti-inflammatory effects of *D. odora* and is one of the main components of the rheumatoid arthritis medication Zushima-Pian [46]. Furthermore, the production of IL-1, TNF-α, and macrophage migration inhibitory factor was significantly reduced by daphnetin in an adjuvant arthritic rat model [46]. Daphnetin has also been extracted from *D. odora* var. *marginata*, was reported as a modulator of Tregs and Th17, and has been suggested as an effective agent for arthritis [47]. Furthermore, Yeşilada et al. reported that daphnetin from *D. oleoides* ssp. *oleoides* had potent anti-inflammatory activity, supporting its folkloric use in rheumatoid arthritis and lumbago [31]. In addition, daphnin (glycosylated daphnetin) showed reduced inhibitory activity against pro-inflammatory cytokines [31].

According to the results of the present study, DJLE significantly suppressed the levels of pro-inflammatory cytokines (IL-1β, IL-6, and TNF-α) and the expression of transcription factors (iNOS and COX-2) involved in the inflammation cascade; however, it was not effective in the antibacterial test (Figure 7). Therefore, these results indicate that although the components of herbal materials (flavan-3-ols [43], for example) have antiperiodontitis effects primarily based on their antibacterial effect, DJLE may have a different mechanism of action in the anti-inflammatory effect. These results strongly suggest that DJLE has both protective and therapeutic effects, making it a strong candidate for periodontitis prevention. Although several studies have been conducted on the anti-inflammatory effects of *Daphne* species, this is the first report demonstrating the chemical profiles and anti-inflammatory properties of DJLE in dental disorders. Based on these results, further in vivo experimentation using animal model is suggested to confirm DJLE’s usefulness in the treatment of periodontitis. Phytochemical isolation from active fractions in *D. jejudoensis* is also needed for screening active components associated with the anti-inflammatory effects. Finally, this study provides evidence for the development of DJLE as a preventive and curative agent for periodontitis and other inflammation-causing diseases, which will be investigated in future studies.

## 4. Materials and Methods

### 4.1. Chemicals

All solvents used for the analysis, acetonitrile, methanol, and formic acid of HPLC grade, were obtained from Fisher Scientific (Hampton, NH, USA). Water for the mobile phase was purified using VSF Elga (Evoqua, Pittsburgh, PA, USA). The ethanol used for the extraction was purchased from Samchun Pure Chemicals (Pyeongtaek, Gyeonggi, Korea). Daphnin was obtained from Sigma-Aldrich (St. Louis, MO, USA).

### 4.2. Plant Materials

**Figure 10:**
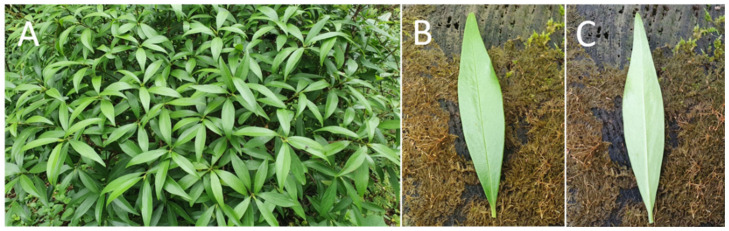
Picture of *Daphne jejudoensis* plant (**A**) and the front (**B**) and back (**C**) of its leaf.

Leaves of *D. jejudoensis* (Figure 10) were collected from the Gotjawal forest area of Jeju Island in May 2021 and authenticated by J.-Y.B. (Jeju National University, Korea). The voucher plant specimen (JNUP-2021-20) was preserved in the herbarium of the College of Pharmacy, Jeju National University.

### 4.3. Sample Preparation

DJLE was prepared by sonication of dried powdered material (10.2 g) using 70% ethanol. Sufficient solvent (ca. 50 mL) was applied to soak the material in three times its volume and extracted for 30 min at 25 °C with vortexing at intervals. The supernatant was collected after centrifugation at 10,000× *g* at 25 °C for 5 min (Avanti J-15R centrifuge; Beckman Coulter, Brea, CA, USA) and filtered through a filter paper. The extraction process was repeated three times, and the pooled supernatant was concentrated using a reduced-pressure rotary evaporator (Heidolph, Schwabach, Germany) at 45 °C. The extract was kept under vacuum overnight for further drying and then harvested for the assay. DJLE was maintained in the dark at 4 °C before the experiment.

### 4.4. Isolation and Cell Culture of hPDLCs

hPDLCs were obtained from ten healthy donors (aged 18–22 years) through third molar extraction and were isolated from the gingival tissue attached to the teeth and periodontal ligament tissue remaining at the root surface of the teeth. All experiments were conducted with the consent of the patients after approval from the hospital’s institutional review board (IRB No: S-D20140007) at the Seoul National University Dental Hospital. The isolated tissues were cut into slices, transferred to 60 mm culture dishes, and incubated in alpha-modified Eagle’s medium (α-MEM; Gibco BRL, Rockville, MD, USA) supplemented with 10% fetal bovine serum (FBS; Gibco BRL, Rockville, MD, USA) and antibiotics under 5% CO_2_ at 37 °C [48].

### 4.5. Cytotoxicity of DJLE on hPDLCs

The cytotoxicity of DJLE was confirmed using the MTT assay (Gibco BRL, Rockville, MD, USA). hPDLCs were grown in α-MEM (Gibco BRL, Rockville, MD, USA) supplemented with 10% FBS (Gibco BRL, Rockville, MD, USA) and antibiotics at 37 °C with 5% CO_2_. The cells were washed with α-MEM/10% FBS medium and seeded onto 96-well plates at a density of 3 × 10^3^ cells/mL. After overnight incubation, the supernatant was replaced and treated with 1, 10, 50, and 100 μg/mL DJLE in media. The MTT assay was conducted after incubation for 0, 24, and 48 h as follows: each well was washed with phosphate-buffered saline (Gibco BRL, Rockville, MD, USA), and MTT solution (5 mg/mL, 50 μL) was applied, followed by 4 h of incubation. After discarding the MTT solution, the formed violet crystals were dissolved in dimethyl sulfoxide (Sigma-Aldrich, St. Louis, MO, USA) and mixed well for homogeneous absorbance measurements at 570 nm (Thermo Max^™^; Molecular Devices Co., San Jose, CA, USA). The control was α-MEM without samples, and all results were calculated as a percentage of the control. The tests were repeated at least three times.

### 4.6. Anti-Inflammatory Effect of DJLE on hPDLCs

hPDLCs were incubated in α-MEM (Gibco BRL, Rockville, MD, USA) supplemented with 10% FBS (Gibco BRL, Rockville, MD, USA), 100 U/mL penicillin (Gibco BRL, Rockville, MD, USA), and 100 μg/mL streptomycin (Gibco BRL, Rockville, MD, USA) at 37 °C in 5% CO_2_ until they were in a single layer. Cells were isolated in a culture dish containing a 10:1:9 mixture of 0.02% ethylenediaminetetraacetic acid (Gibco BRL, Rockville, MD, USA):2.5% trypsin:phosphate-buffered saline (Gibco BRL, Rockville, MD, USA). The cells were incubated in a 60 mm culture dish at a density of 2.5 × 10^5^ cells/mL, followed by washing with media (α-MEM/10% FBS). The medium was replaced after overnight incubation, and then, the experiment for anti-inflammatory effects was performed as follows: (1) sham control, (2) LPS treatment alone, for the negative control (1 μg/mL, inflammatory induction), (3) DJLE single treatment (50 μg/mL), (4) LPS (1 μg/mL) + DJLE (50 μg/mL) (for the evaluation of the preventive effect), and (5) LPS (1 μg/mL, 24 h pretreatment) + DJLE (50 μg/mL) (for the assessment of treatment effects). After 48 h, the expression of inflammatory factors was analyzed by measuring the mRNA levels under the influence of DJLE in comparison to those in the control.

### 4.7. Reverse Transcription–Polymerase Chain Reaction and Real-Time Polymerase Chain Reaction Analyses

Total RNA from hPDLCs was isolated using the TRIzol reagent. Complementary DNA synthesis was performed using 2 μg of total RNA: 1 μL of reverse transcriptase and 0.5 μL of oligo dT (Invitrogen, Thermo Fisher, Waltham, MA, USA). The cDNA synthesized from hPDLCs was subjected to real-time PCR using the primers listed in Table 1. Real-time PCR was performed on an ABI PRISM 7500 sequence detection system using SYBR Green PCR Master Mix (Takara, Tokyo, Japan), according to the manufacturer’s instructions. The PCR conditions were 94 °C for 1 min, followed by 40 cycles of 95 °C for 15 s and 60 °C for 34 s. All experiments were performed in triplicate and normalized to the expression of the housekeeping gene, *GAPDH*. The results were analyzed using the comparative cycle threshold (CT) method. The primers used for real-time PCR are listed in Table 1. 

### 4.8. Antimicrobacterial Effect of DJLE on Porphyromonas gingivalis

*Porphyromonas gingivalis* was obtained from the Korean Collection of Oral Microbiology (KCOM), School of Dentistry, Chosun University. The DJLE was diluted to different concentrations (from 0.01 to 0.1 mg/mL) and prepared with chlorhexidine (10 μM, Daewoong Pharm, Seoul, Korea) in tryptic soy broth (TSB; BD DIFCO, USA) to a total volume of 1 mL for each tube. *P. gingivalis*-strain-containing media (100 μL) were inoculated to obtain a density of 1.0 × 10^4^ CFU/mL. The tubes were incubated for 24 h at 37 °C, and turbidity was observed. Bacterial growth was monitored by measuring OD600 using a microplate reader (BioTek Instruments, Winooski, VT, USA). The minimal inhibitory concentration was defined as the lowest DJLE concentration at which no bacterial growth was detected. The tests were repeated at least three times.

### 4.9. Chemical Profiling of DJLE and Isolation of a Major Compound

A liquid chromatographic system (Agilent series 1290) consisting of a binary pump, a solvent degasser, a 100-well tray auto-sampler, and a temperature-controlled column compartment (Agilent Technologies, Santa Clara, CA, USA) was used to identify the chemical components of DJLE. Separation was performed at 40 °C using an Eclipse Plus C18 RRHD 1.8 μm column (100 × 2.1 mm, Agilent Technologies, Santa Clara, CA, USA) with a 3 μL injection volume. The mobile phase consisted of 0.1% formic acid in water (A) and 0.1% formic acid in acetonitrile (B) in gradient mode as follows: 0–1 min, 5% B; 1–4 min, 5%–60% B; 4–7 min, 60%–98% B; 7–9 min, 98% B. The flow rate was 0.3 mL/min. Daphnin was isolated on a 100 g cartridge filled with silica gel using methylene chloride and methanol as the mobile phase in medium-pressure liquid chromatography (Isolera; Biotage, Uppsala, Sweden) at the Bio-Health Materials Core-Facility, Jeju National University and further purified using preparative HPLC (1260 Infinity II series, Agilent Technologies, Santa Clara, CA, USA). The reference compound daphnin was purchased from Sigma-Aldrich and compared in terms of retention time with the isolated compound for confirmation. The molecular formulas and chemical structures of the other compounds were screened using high-resolution mass spectrometry (Agilent Model no. G6546A Q-ToF-MS/MS, Agilent Technologies, Santa Clara, CA, USA) equipped with an ESI source with Jet Stream technology using the following parameters: drying gas, N_2_; flow rate, 8 L/min; drying gas temperature, 300 °C; nebulizer, 40 psi; sheath gas temperature, 350 °C; sheath gas flow, 11 L/min; capillary, 3500 V; skimmer, 65 V; octopole radiofrequency (Oct RF), 750 V; fragmentor voltage, 150 V. DJLE was analyzed in the range of *m*/*z* 100–1000 in negative mode. Reference ion correction using reference masses at *m*/*z* 112.9856 (deprotonated trifluoroacetic acid, TFA) and *m*/*z* 1033.9881 (TFA-adducted HP-921) was used in the negative ion electrospray for accurate mass measurements. Mass Hunter Qualitative Analysis software (version 10.0) was used to process the mass data containing molecular feature extraction, background subtraction, and molecular formula estimation.

### 4.10. Data Analysis

The results are presented as mean ± standard deviation of triplicate experiments. The statistical significance of the differences among the samples was evaluated using one-way analysis of variance (ANOVA). The values were considered statistically significant at *p*-values less than or equal to 0.05 compared with the control.

## 5. Conclusions

In summary, our results demonstrated that DJLE has inflammation-modulating effects in periodontal disease, having both preventive and therapeutic effects. This is the first report to show the chemical profiles and anti-inflammatory properties of *D. jejudoensis.* Our findings suggest that the anti-inflammatory properties of 70% ethanolic extract from *D. jejudoensis* leaf might be due to the inhibition of IL-6, IL-1β, TNF-α, iNOS, and COX-2 expression. Moreover, these results provided evidence to develop DJLE as a therapeutic agent for periodontitis as well as other diseases caused by inflammation.

## Figures and Tables

**Table 1:** Nucleotide sequences of real-time PCR primers.

Gene	Primer (5′-3′)	
*hTNF-α*	forward	CCCAGGGACCTCTCTCTAATC
	reverse	ATGGGCTACAGGCTTGTCACT
*hIL-1β*	forward	ACGCTCCGGGACTCACAGCA
	reverse	TGAGGCCCAAGGCCACAGGT
*hIL-6*	forward	AGGAGACTTGCCTGGTGAAA
	reverse	GCATTTGTGGTTGGGTCAGG
*hiNOS*	forward	GTTCTCAAGGCACAGGTCTC
	reverse	GCAGGTCACTTATGTCACTTATC
*hCOX2*	forward	TTCTCCTTGAAAGGACTTATGGGTAA
	reverse	AGAACTTGCATTGATGGTGACTGTTT
*hGAPDH*	forward	CCATGGAGAAGGCTGGGG
	reverse	CAAAGTTCTCATGGATGACC

## Data Availability

Data is contained within the article.
